# New classifications of axillary lymph nodes and their anatomical-clinical correlations in breast surgery

**DOI:** 10.1186/s12957-021-02209-2

**Published:** 2021-03-29

**Authors:** Roberto Cirocchi, Maria Ida Amabile, Alessandro De Luca, Federico Frusone, Domenico Tripodi, Patrizia Gentile, Renata Tabola, Daniele Pironi, Flavio Forte, Massimo Monti, Vito D’Andrea, Salvatore Sorrenti

**Affiliations:** 1 Department of Surgical Science, University of Perugia, Perugia, Italy; 2 Department of Surgical Sciences, Sapienza University of Rome, Viale Regine Elena 324, 00161 Rome, Italy; 3 Department of Gastrointestinal and General Surgery, Medical University, Wroclaw, Poland; 4 Urology Department, M.G. Vannini Hospital, Rome, Italy

**Keywords:** Axillary lymph node, Clough’s classification, Li’s classification, Sentinel lymph node, Breast cancer, Breast surgery

## Abstract

**Background:**

In the last decade, two research groups, the French group by Clough et al. (Br J Surg. 97:1659–65, 2010) and the Chinese one by Li et al. (ISRN Oncol 2013:279013, 2013), proposed two types of classification of axillary lymph nodes in breast cancer, identifying novel anatomic landmarks for dividing the axillary space in lymph node dissection.

**Main body:**

Knowledge of the exact location of the sentinel node helps to focus the surgical dissection and to reduce the morbidity of sentinel lymph node biopsy procedures, in particular the risk of arm lymphedema, without compromising sensitivity.

**Conclusion:**

In this article, we aimed at focusing on the clinical impact that the most recent classifications of axillary lymph nodes have obtained in literature, highlighting the importance of defining new demarcations to preserve the axillary lymph nodes as much as possible in breast surgery.

## Introduction

**Fig. 1:**
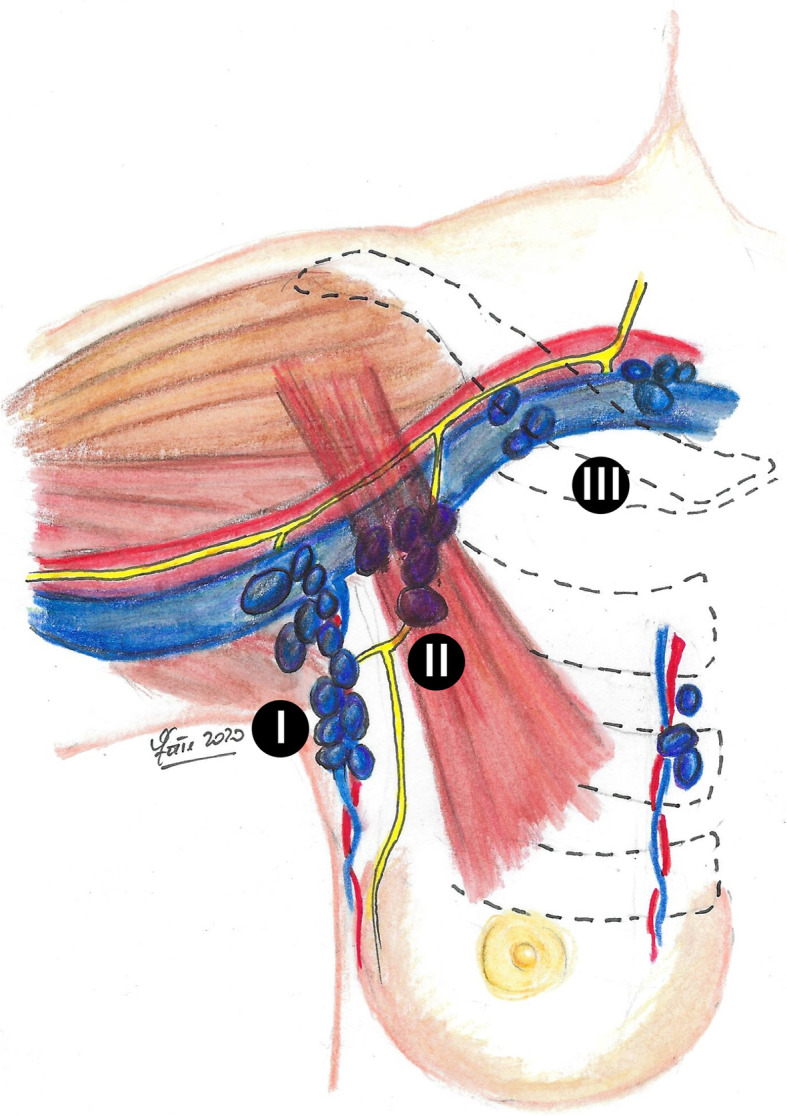
The anatomical classification of the axillary lymph nodes (I level, lymph nodes located lateral to the lateral margin of the minor pectoralis muscle; II level, lymph nodes located between the medial and lateral margin of the minor pectoralis muscle and the interpectoral lymph nodes; III level, lymph nodes located medial to the medial margin of the minor pectoralis muscle)

The anatomical classification of the axillary lymph nodes has undergone over time variations, particularly related to their clinical implications (Fig. 1) [1]. Attention has been paid to lymph node dissection and lymphedema, which represents the most disabling and often permanent complication after axillary dissection [2]. Upper limb lymphedema occurs in a very variable percentage of patients undergoing axillary surgery (7–77%) [3], and it is for this reason that some authors have proposed new classifications to minimize the axillary lymph node removal and therefore reduce the risk of lymphedema [4, 5].

The interest in this research topic stems from the fact that breast cancer is the most common malignancy in women in the western countries [6, 7]. Until recently, the main treatment for this tumor was radical mastectomy accompanied by axillary lymph node dissection (ALND) in order to obtain optimal control of the locoregional tumor and achieve long-term survival [8].

Although in the late 1990s the introduction of sentinel lymph node (SLN) biopsy developed a minimally invasive procedure for staging breast cancer thus reducing the use of ALND, a significant percentage of breast cancer patients still undergo primary or completion ALND for tumor staging and optimization of locoregional control [9, 10].

Functional studies on lymph drainage of the upper limb [1, 11, 12] have identified two distinct lymphatic drainage pathways in the axillary region, one medial of the breast and one more lateral of the arm, which can be distinctly preoperatively marked by intradermal injections of radioisotope or vital dye into the breast and arm.

If upper limb lymphedema is caused by interruption of the corresponding axillary lymphatics and/or removal of the lymph nodes, the possibility to identify and preserve them might prevent upper limb lymphedema during axillary surgery.

In this light, the axillary reverse mapping, a procedure developed to allow the identification of the lymphatic drainage path of the arm in order to save lymph nodes [11, 12], has attracted more attention, and several studies have reported the clinical utility of this possible improvement of the ALND technique in an attempt to reduce morbidity while preserving its oncological safety.

Moreover, in the following years, two research groups, the French and the Chinese ones, proposed two types of classification of axillary lymph nodes [1, 13].

### Aim of the study

The main objective of our work was to highlight the clinical impact that the most recent classifications of axillary lymph nodes have obtained in literature. In this way, the paper aims to understand the importance of defining new and safe demarcations to preserve the axillary lymph nodes as much as possible in breast surgery.

## Clough’s classification

**Fig. 2:**
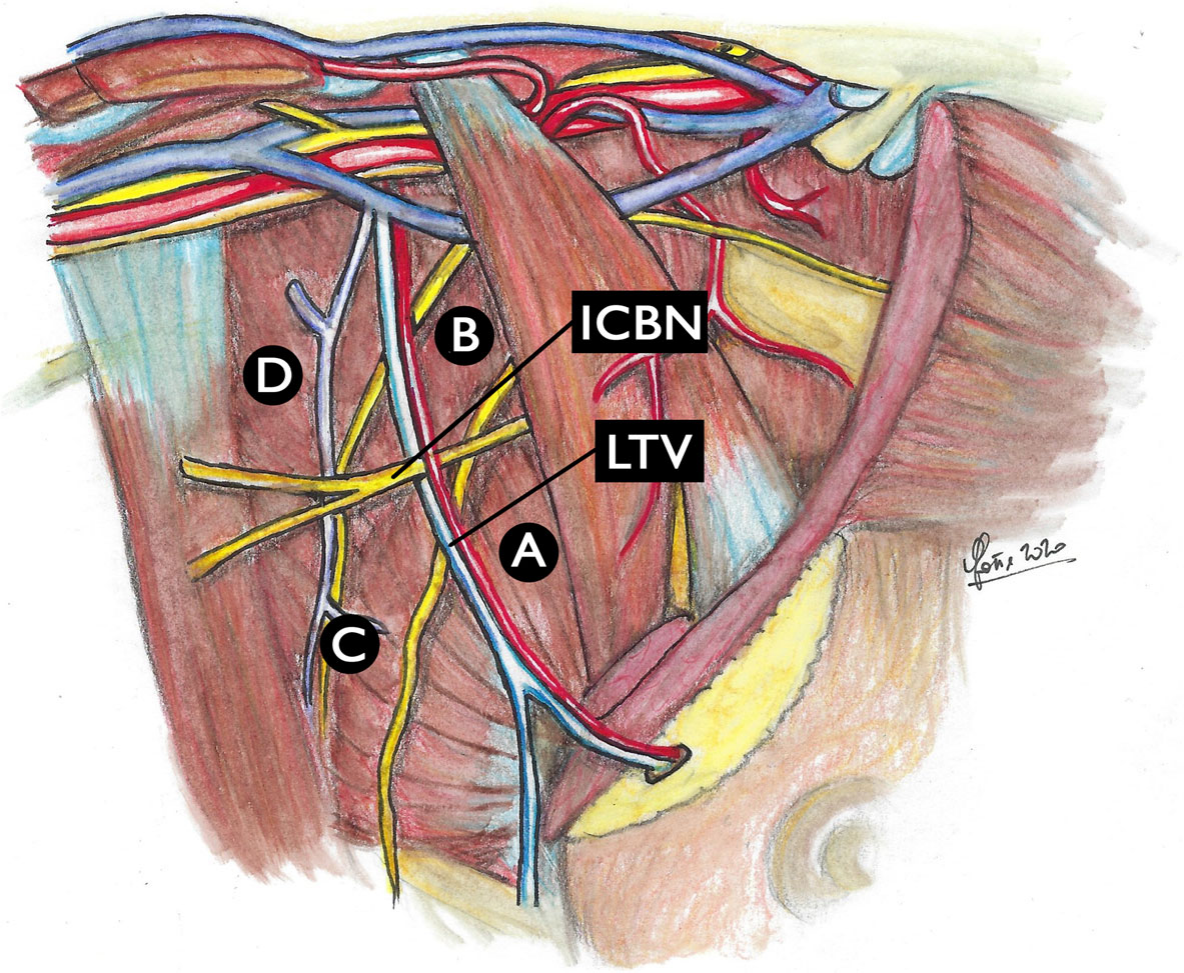
Clough’s classification of axillary lymph nodes. The intersection between the lateral thoracic vein (LTV) and the second intercostobrachial nerve (ICBN) forms a cross that creates 4 zones: zone **a** is the area adjacent to the LTV, extending from the lower border of the axilla to the second ICBN; zone **b** is the area adjacent to the LTV, extending from the second ICBN to the axillary vein, extending medially under the pectoralis minor muscle; zone **c** is the lateral axillary area outside zone **a**; and zone **d** is the lateral axillary area outside zone **b**

In 2010, a French research group by Clough et al. proposed and described a new anatomical classification of the lower axilla, based on the intersection of two anatomical structures, the second intercostobrachial nerve (ICBN) and the lateral thoracic vein (LTV). The purpose was to identify the position of the axillary SLN and to demonstrate its non-random position, as well as to confirm that the breast lymph drainage follows a predetermined pathway (Fig. 2) [1].

The intersection between the second ICBN (horizontally) and the LTV (vertically) is a constant anatomical finding in this region, forming a cross delimiting 4 zones: zone A is the area that extends from the lower margin of the axilla to the second ICBN; zone B is the area that extends from the second ICBN to the axillary vein under the pectoralis minor muscle; zone C is the lateral axillary area external to zone A; and zone D is the lateral axillary area external to zone B.

The study included more than 200 patients with stage I breast cancer or ductal carcinoma in situ undergone SLN identification in the axilla by peritumoral injection. Clough et al. documented that in around 98% of patients the axillary SLN was located medially to the LTV, either in zone A below the second ICBN or in zone B above it [1]. The authors documented that this classification may be able to avoid unnecessary more lateral axillary dissections and useful for surgeons when performing SLN biopsy procedures in order to identify its precise location. In this light, knowledge of the exact position of the SLN should in fact help to focus the axillary dissection and reduce the morbidity of the SLN biopsy procedures without compromising the upper limb sensitivity.

## Li’s classification

**Fig. 3:**
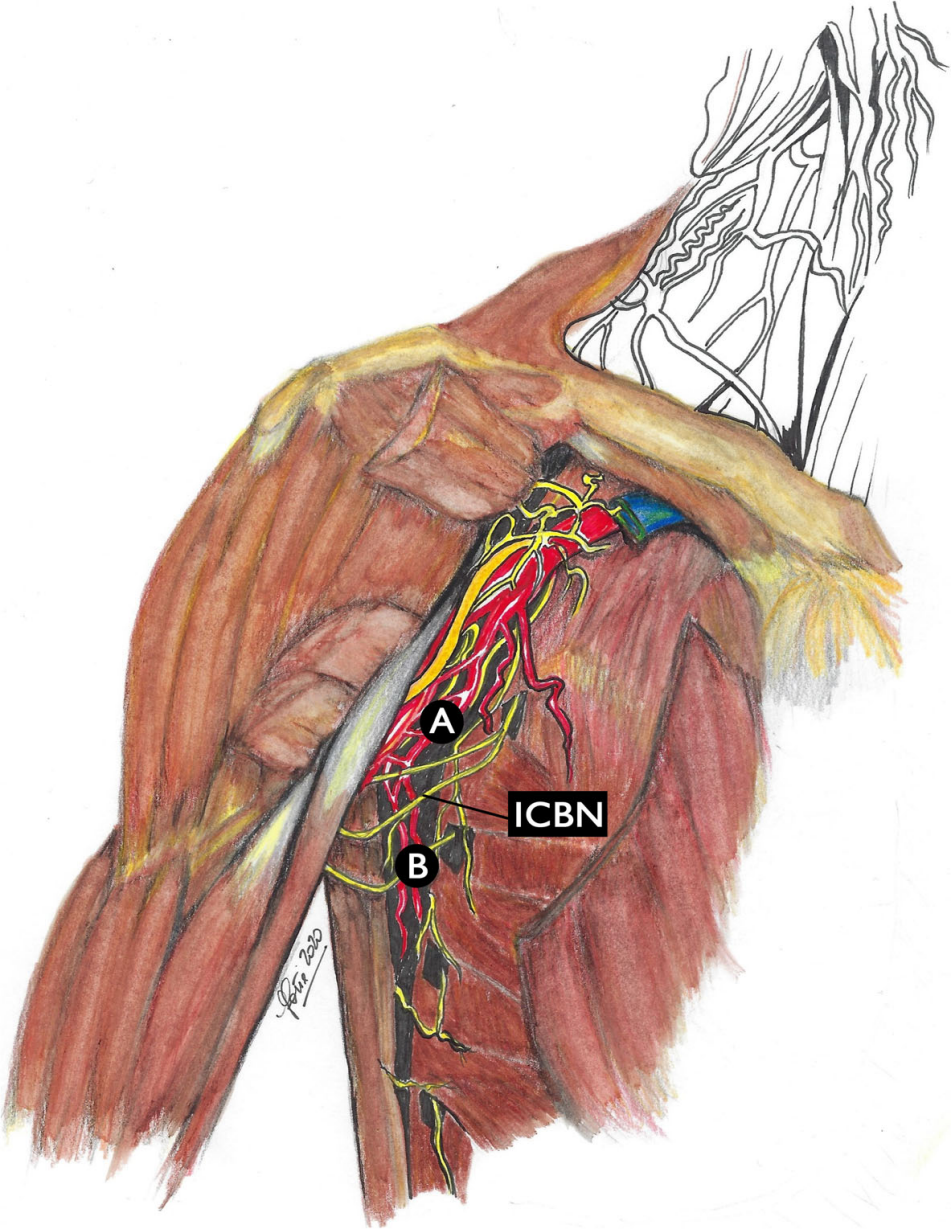
Li’s classification of axillary lymph nodes. The second intercostobrachial nerve (ICBN) divides the axillary space into a lower part (**b**) and an upper (**a**) part

In 2013, a Chinese research group lead by Li et al. conducted a study with the aim of evaluating the use of the second ICBN as a possible new anatomical landmark for the axillary lymph node dissection in breast cancer patients [13]. The authors considered to divide the axillary space by the ICBN into an upper part (A) and a lower part (B) (Fig. 3).

The authors enrolled 72 breast cancer patients undergoing ALND and SLN biopsy procedure [13]. The lymph nodes were divided in 2 groups: those of the upper axillary space and those of the lower axillary space, relative to the ICBNs. All lymph nodes were analyzed to identify macro- and micrometastasis. All the SLNs were found under the ICBNs, and more than ten lymph nodes were located in that space (zone B) [13]. Moreover, when lymph nodes macrometastasized or micrometastasized above the ICBNs, the authors also observed metastasis-positive lymph nodes under the nerves (zone B); when the lymph nodes in zone B showed no metastasis, the lymph nodes over the ICBNs were also not metastatic. In this light, the authors documented that intercostobrachial nerves might be considered potential candidates for a new anatomic landmark to be used in procedures of lymph node dissection [13].

## Clinical applications of axillary Clough’s and Li’s classifications

We conducted a review of the literature using PubMed database to identify original articles that assessed the role of axillary lymph nodes’ classification in breast cancer axillary management.

The following terms or key words were used: “breast cancer surgery”, “lymphatic arm drainage”, “lymphedema”, “Clough’s classification”, and “Li’s classification”. The research was limited to articles published in English and performed on humans. We investigated the results of prospective and non-prospective clinical studies which considered Clough’s and Li’s lymph node classifications with the aim of performing a critical evaluation and verifying the practical and surgical impact that these classifications have obtained after their publication. In particular, we analyzed the clinical contribution that the two research groups offered to surgery to limit the devastating consequence of lymphedema. With the exception of the studies by Clough et al. [1] and Li et al. [13], no other study truly evaluated the feasibility of their techniques’ effectiveness and was able to support the clinical applicability.

In 2010, the research group by Clough et al. [1] described and proposed in the clinical practice a new anatomical classification of the axillary region divided into four areas. Of the other studies that cited this classification, none took advantage of the same demarcation and was able to support its truthfulness. Some authors, although interested in Clough et al.’s indications, said that surgical treatment of the axillary region should be personalized based on the involvement of the lymph node status and did not report any data regarding the lymph node area involved [14, 15].

Li et al., after having investigated the metastasis patterns of lymph nodes and distribution of axillary lymph nodes in relation to the ICBN which was found to be an anatomic marker dividing the axillary space into superior and inferior parts [13], referred to their proposed anatomic demarcation in a subsequent study and found that in all the patients with up to 2 positive nodes the nodes were located inferior to the ICBN, and the incidence of lymphedema for SLN biopsy and partial axillary dissection was 0% [16].

The main problem we encountered was that the various analyzed studies did not specifically describe the exact drained axillary region.

In addition, during preservation of the axillary lymph nodes that drain the upper limb, there is the risk of compromising oncological safety of the surgery. The ARM procedure, based on the assumption that the lymphatic pathway from the upper extremity would not be affected by metastasis from the primary breast cancer, was developed with the aim of reducing lymphedema rates by preserving lymphatic drainage of the upper limb during SLN biopsy and ALND [17]. However, questions remain regarding whether preservation of these lymph nodes affects oncological risk.

Studies have reported the metastatic rates to the ARM nodes in a range from 14 to 43%, indicating a possible connection between the lymphatic drainage pathways of the breast and upper limbs [18, 19], and found that none of the metastatic lymph nodes corresponded with the SLN [17, 20].

Furthermore, special attention should be paid also to the possible existence of a crossover between breast SLNs and ARM lymph nodes, which may make impossible to preserve the arm-draining lymph nodes. Studies investigating the SLN biopsy and ARM procedures reported that patients with concordance between the SLN and ARM nodes during SLN biopsy procedure had a higher lymphedema occurrence rate than those without node concordance [17, 21]. These results could suggest that the connection between the lymph drainage pathways of the breast and extremities would lead to lymphedema even in patients who undergo only SLN biopsy.

Moreover, authors have investigated the ARM technique applied during SLN biopsy and found a considerably lower identification rate for upper limb lymph nodes, considering the procedure insufficient in some cases [22–24]. This difficulty may be mainly due to the location of the upper limb’s SLN, which in most cases is situated below or at the level of the second ICBN, making it difficult to be identified during the SLN biopsy procedure [21].

## Do new axillary demarcations preserve the axillary lymph nodes as much as possible in breast surgery?

The advent of SLN biopsy procedure has changed the axilla’s surgical management in breast cancer patients, avoiding ALND in selected patients [25]. However, for those patients with clinically positive axillary lymph node, ALND still represents the standard of care, although associated with substantial morbidity, including upper limb lymphedema, potential shoulder dysfunction and discomfort, and numbness [26–28].

It has been hypothesized that an altered lymphatic drainage in the axilla is responsible for the increased lymphedema rate [11, 29]. Considering that the upper limb and the breast have their own pathways of lymphatic drainage and that breast cancer may determine an increased lymphatic flow, these aspects have led physicians to investigate the significance of locoregional lymphatic tissue management, as well as to optimize the axillary lymph node metastases mapping as a prognostic factor [30].

After the publication of the results of the ACOSOG Z001120 study and the diffusion of the American Society of Clinical Oncology clinical practice guidelines [31, 32] documenting the low usefulness of ALND even in the presence of metastases in the SLN, the traditional concept of axillary dissection in breast cancer has become an issue to be questioned. However, while the role of axillary ultrasound in the preoperative staging and in the follow-up after SLN biopsy procedure and the possibility of avoiding it are currently being evaluated in a prospective study, SLN biopsy remains the gold standard for the axillary management for early-stage breast cancers [15].

The study performed by Clough et al. [1], conducted with the aim to map the SLN location related to axillary anatomic landmarks, found that 98% of the breast-draining lymphatics were medial to the LTV, suggesting that the first breast-draining lymph nodes may be more medial than originally appreciated. Moreover, the authors hypothesized that lymph nodes lateral to the LTV were more specific to the lymphatic drainage of the upper limb than to the drainage of the breast gland [1]. This may in part explain why exclusion of the upper-level axilla from the radiation field after ALND may limit lymphedema rates [33]. Karampelias et al. [30], driven by Clough’s classification, tried to link the topographical position of SLN to the pathological positive result for metastasis and documented that the majority of the positive lymph nodes are located in the front axillary region.

Although nowadays postoperative upper limb lymphedema occurs in a minority of breast cancer patients, and in particular cases such as inflammatory breast cancer [34], its development markedly reduces the quality of life. Upper limb lymphedema may be caused by the resection of the lymph nodes and lymphatic vessels during ALND and also during up to 7% of SLN biopsy procedure [15], in particular when a greater number of non-SLNs are removed to reduce the false-negative rates during this procedure. In this light, the greater the number of resected non-SLNs, the greater the probability of occurrence of upper limb edema [3]. Accuracy in identifying and removing SLN may limit this event. Specifically, it is important to correctly map SLNs, identifying areas often overlooked during surgery as well as factors related to inappropriate skin incisions, in order to eliminate those conditions that may make difficult the SLN dissection and may increase rate of false negative SLN [14]. In addition, this may provide useful guidance for training of unexperienced surgeons and to reduce the risk of postoperative lymphedema.

Only few studies had described the distribution pattern of SLNs in axillary tissues [1, 13, 35]. Clough et al. [1] documented that the majority of SLNs were located at the medial part of the axilla, alongside the LTV. Li et al. [13] found all the SLNs under the ICBNs. Interestingly, a study showed that SLNs were mainly found between the pectoralis major muscle and the LTV; nevertheless, this study used only the anatomical method to identify SLN, and the false negative rate was up to 22% [35]. Taking into account that these studies present limitations, they all confirmed that the distribution of SLNs was different and that if it is paid attention only to certain axillary areas, the false negative rate of SLN biopsy would increase [36]. Moreover, these findings may help to avoid lymphedema caused by unnecessary dissection of the axilla in the area lateral to the LTV and upper the ICBN, also allowing easier identification of the SLN.

In addition, although axillary management for early breast cancer with micrometastatic SLN is well recognized, there is still no consensus in case of macrometastatic SLN involvement. It is pivotal to distinguish between two different aspects: (i) further axillary surgery versus no further surgery; (ii) axillary surgery versus axillary irradiation [37–39]. In particular, this evolving modification in axillary surgery determines a crucial redefinition of the role of radiotherapy in patients with macrometastatic SLN who did not receive ALND [37–39].

Nevertheless, several issues remain to be solved, including the oncological safety of arm lymph node preservation and the real effectiveness of reducing arm lymphedema. Although a correct axillary demarcation may preserve the axillary lymph nodes as much as possible in breast surgery, patients with higher BMI and increased axillary disease burden and axillary radiation may have higher risks of developing arm lymphedema. Further clinical studies are needed for developing an easy identification procedure to detect SLN location. This will eliminate the problem of false negativity in SLN mapping. Moreover, such information could be useful to reduce the morbidity of SLN biopsy procedure and to increase its reliability, representing an adjunct for practicing and shortening the learning curve required for a breast surgeon.

## Data Availability

All data analyzed during this review study are included in this article.
